# Multilayer radiation shield for satellite electronic components protection

**DOI:** 10.1038/s41598-021-99739-2

**Published:** 2021-10-19

**Authors:** Hamideh Daneshvar, Kavoos Ghordoei Milan, Ali Sadr, Seyed Hassan Sedighy, Shahryar Malekie, Armin Mosayebi

**Affiliations:** 1 Radiation Application Research School, Nuclear Science and Technology Research Institute, Tehran, Iran; 2 School of Advanced Technologies, Iran University of Science and Technology, Tehran, Iran; 3 School of Electrical Engineering, Iran University of Science and Technology, Tehran, Iran; 4 Radiation Application Research School, Nuclear Science and Technology Research Institute, Karaj, Iran

**Keywords:** Aerospace engineering, Electrical and electronic engineering

## Abstract

In this paper, various multi-layer shields are designed, optimized, and analyzed for electron and proton space environments. The design process is performed for various suitable materials for the local protection of sensitive electronic devices using MCNPX code and the Genetic optimization Algorithm. In the optimizations process, the total ionizing dose is 53.3% and 72% greater than the aluminum shield for proton and electron environments, respectively. Considering the importance of the protons in the LEO orbits, the construction of the shield was based on designing a proton source. A sample shield is built using a combination of Aluminum Bronze and molybdenum layers with a copper carrier to demonstrate the idea. Comparisons of radiation attenuation coefficient results indicate a good agreement between the experimental, simulation, and analytical calculations results. The good specifications of the proposed multi-layer shield prove their capability and ability to use in satellite missions for electronic device protection.

## Introduction

Space radiation is one of the most important issues in the design of space systems. The electronic equipment used in the satellite missions is encountered with ionizing particles, which may cause some problems in their normal operation^1^. The radiation damage in the electronic equipment can be divided into three categories: total ionizing dose (TID), displacement damage (DD), and single event effect (SEE)^2^.

Some countermeasure strategies should be considered to successfully perform a space mission in designing space systems to deal with radiation damage. One of the most effective solutions is using the appropriate shielding to protect sensitive electronic parts. These shields should be designed by considering the satellite mass and volume budgets^3^. The shielding material type, optimal shielding thickness, material type, and sorting of the shielding layers can be varied depending on the desired radiation environment. It should be mentioned that lightweight materials cannot efficiently attenuate the energetic electrons and protons, and heavy materials can create secondary particles, so the combination of high-density shielding materials such as tantalum and tungsten and low-density ones such as polyethylene can be considered as an ideal strategy^4–6^. There are some programs for transporting radiation particles in the shielding materials through the Monte Carlo methods, such as the MCNP code^7–23^.

The satellite structure is the first radiation shield layer where its weight, vibration tolerance, natural frequency range and ability to withstand against space radiation should be considered in its design process. In the first level of protection, the satellite structure absorbs all or some of the emitted flux, depending on its material and thickness. In the second level, holder boxes named local shields, metal boxes containing electronic boards and sensitive equipment are used^24^. Based on the existence of weight and vibration tolerance limitations in the construction of satellite structures, the most percentage of radiation protection is done by using holder boxes. In order to reduce the secondary effects of impact particles, multi-layer shields are used. Space is complicated environment and contains a variety of particles, and there is no laboratory practically to simulate such a complex environment on the Earth. Therefore computational methods are effective tools to design radiation shields. The importance of this issue is result in the reliability enhancement of the satellite system, reducing operating costs and project risks^3,25^.

The basis of this research is based on the design of radiation shield for using in LEO satellites. Trapped electrons and protons have the largest contribution of space radiation in LEO orbits. Therefore, the MCNPX code simulation is performed by using these two types of particles, electrons and protons in the worst-case scenario. Photons are also the most penetrating particles, so in experimental experiments, gamma rays are used to estimate the worst conditions, also^26,27^.

In the second and third sections of the paper, a multi-layer radiation shield structure is designed by using Genetic algorithm (GA), to protect the electronic devices against the electrons and protons in the space environments especially in the LEO ones. The type of materials and shield layer thicknesses are two set parameters that should be designed to achieve the best protection against space radiation. In this work, random numbers are generated via GA for these two set parameters. The MCNPX code analyses the shield structure according to them, where its outputs include ionization dose, mass and secondary particles. These outputs are added according to their importance via cost function, and imported in MATLAB to be used in the optimization process. This process iterates until the optimization converge into the best thickness and material based on the output dose and secondary particles. In fact, these two set parameters (material type and thickness) are optimized by using GA in electron and proton space environments^28–31^ by linking MCNPX code with MATLAB software. In the fourth section, a simple three-layer radiation shield is fabricated and tested by using commercial off-the-shelf (COTS) available materials for demonstration.

The designed radiation shields for electrons and protons space environments exhibited 53.3% and 72% shielding effectiveness, respectively, rather than an aluminium shield with the same thickness. Finally, a three-layer radiation shield with 2 mm thickness is designed and implemented based on the commercial off-the-shelf (COTS) available materials. The good protection ability of the proposed structure candidates it for space application to protect the electronic devices.

## Multi-layer structure design

There are two important points in the shield design for space systems that are placed in front of the charged particles: reduction of secondary particles due to the collision of charged particles with the shield material, and reduction of the received dose of the subsystems. These two factors can be controlled according to nature of the charged particles and it is investigated in this article. Due to higher penetration of photons than charged particles, the quantity of “attenuation coefficient” is considered to design of shields, which is discussed below^32,33^.

**Figure 1:**
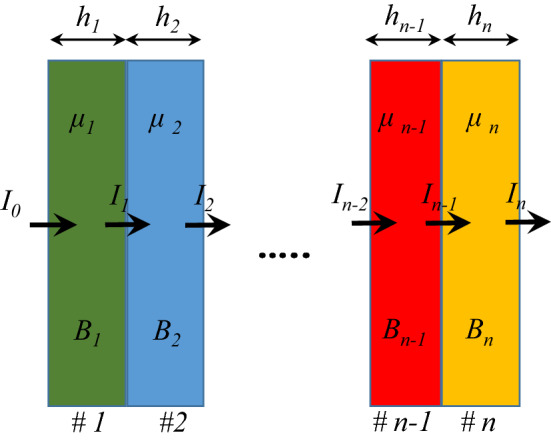
Schematic of multi-layer radiation shield.

The schematic of the multi-layer structure is presented in Fig. 1. When a shield layer is placed in the front of a photon source, such as X- and gamma rays, for the low thickness of the absorber and narrow or well-collimated beam, the gamma-ray flux follows the Beer-Lambert equation^16,17,23^ as

1 $$ I = I_{0} e^{{ -\upmu {\text{x}}}} $$

In which I is the intensity of the rays after passing through the shield, I0 is the intensity of the initial rays, x is the shield thickness, and μ is the radiation attenuation coefficient. For wide beams, the build-up factor, B is added to this equation for correction as

2 $$ I = BI_{0} e^{{ -\upmu {\text{x}}}} $$

In the case of multi-layered shields, the intensity of the source passing through these multiple layers follows the equation as^34,35^.

3 $$ I = B_{1} B_{2} B_{3} I_{0} e^{{ - (\upmu _{1} {\text{x}}_{1} +\upmu _{2} {\text{x}}_{2} +\upmu _{3} {\text{x}}_{3} )}} $$

Various analytical and simulation methods can be used to determine the radiation attenuation coefficient of multilayer protections, μ. However, using transport methods to determine this coefficient can only be performed for simple geometries used in several similar articles for different applications^18,23,36^. Here, the MCNPX code and MULASSIS tool are used to determine the radiation attenuation coefficient in simulation^16–20,37–39^. Finally, XCOM software is also used to determine the attenuation coefficient as an analytical method. Using these three programs, it is possible to validate the results.

Table 1 provides some of the materials that have been reported in the literature data for using in radiation shield design^4,40^. This table contains eight pure materials that are very common for shield design and used to design and optimize the proposed multi-layer radiation shielding structures.

**Table 1:** Common materials used for designing the radiation shields.

Number	Material	Density (g/cm^3^)
1	Tantalum	16.69
2	Tungsten	19.25
3	Lead	11.34
4	Aluminium	2.7
5	Silver	10.49
6	Gold	19.30
7	Copper	8.94
8	Titanium	4.5

**Figure 2:**
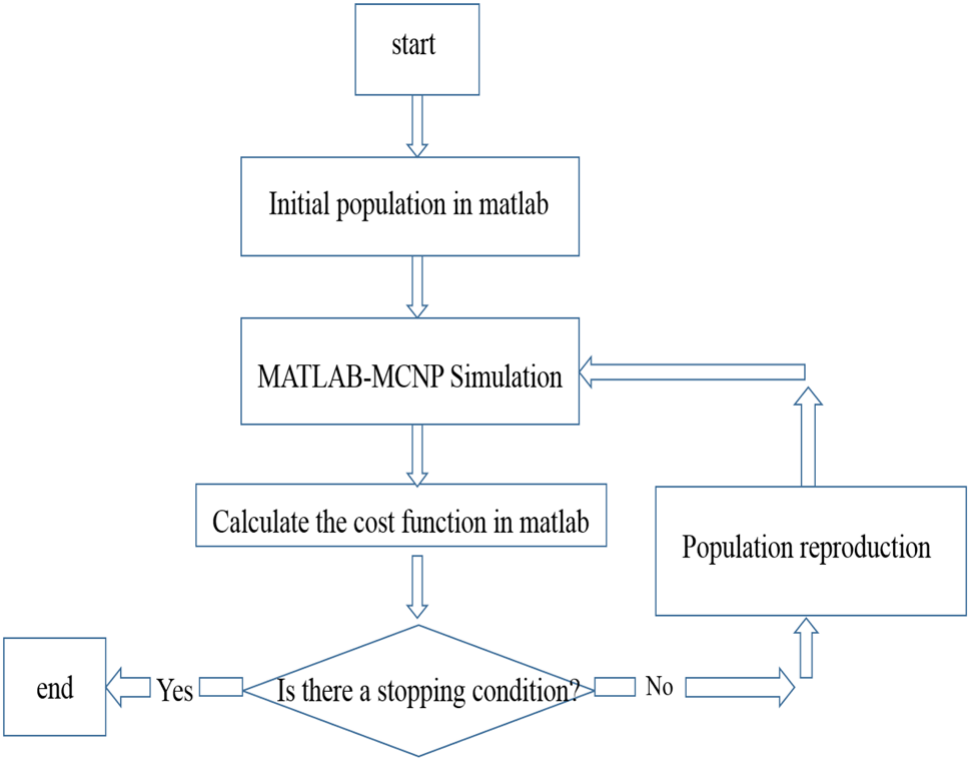
Flowchart of the Genetic Algorithm design process.

The cost function for optimization is defined as

4 $$\mathit{Cos}t=\alpha \times TID+\beta \times SP$$

where the sum of total ionizing dose (TID) and the secondary particles (SP) are added together with appropriate weighting coefficients, α, and β. “TID and SP calculations are performed using MCNPX code. The radiation dose is defined as the energy deposited in the material, where its unit is the ratio of energy to the mass of material (J/g). To achieve optimal shield, the combination of these two parameters in (4) should have the lowest cost, and therefore an optimization method would be used. The optimization method is the Genetic Algorithm, so the MCNPX code is linked to MATLAB software for optimization implementation”. The output of this optimization is the thickness of each layer as well as its material. The optimization flowchart in Fig. 2 depicts how to implement this design process.

The combination of the MCNPX code and MATLAB software is used to design the optimal shield. Also, the experimental method is used to validate the shield design. Based on this design, the shield is built and placed in the front of source for radiation testing. Before and after shield placement in the front of source, its flux is measured, and the radiation attenuation coefficient is obtained by using (1). In addition to the experimental method, the radiation attenuation coefficient is calculated by using MCNPX and MULASIS computational codes and XCOM software. The next step is comparison between the radiation attenuation coefficient resulted from experimental and computational methods. If the results are close to each other, it can be concluded that the work with the MCNP code is done correctly and subsequently, it can be concluded that the results of the shield design using the combination of MCNPX code and MATLAB have the necessary accuracy and validity. Notice that the electrons and protons spectrum of a common LEO satellite are considered in simulation. In fact, we aim to design a general effective multi layer shield instead of specified one in a predetermined satellite.

## Different shields design

**Figure 3:**
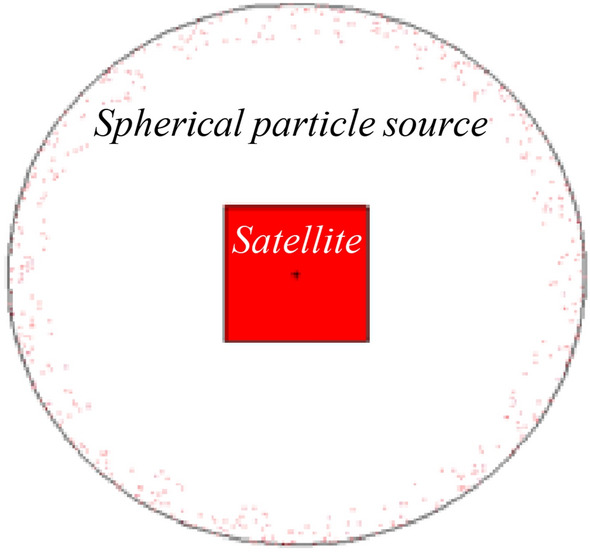
2D display of the problem structure with a spherical source.

The satellite platform is considered cube-shaped, with dimensions of 1 × 1 × 1 m^3^ in the MCNP simulation process. The worst radiation orbital conditions are also considered to design an effective shield in all orbits. For this purpose, the source is considered as a single particle spherical around the satellite. The general structure for the satellite is shown in Fig. 3, which is simulated by the MCNPX code. In fact, the MCNPX code evaluates and achieves the cost function of the GA proposed population through the generations via the link between MATLAB and MCNPX automatically.

### Low dose environment

Now, we focus on the analysis and optimization of radiation shielding for electron environments. These optimizations are performed for three types: three, five, and seven-layer shields. The shields are taken into account under the worst space conditions and optimized for electron environments, including electrons with the energy spectrum in the range of 1–25 MeV^6^. A single-particle source is considered in which it emits electrons toward the satellite. Damage on satellite electronic is produced by total ionizing dose and secondary particles. To minimize the damage induced by the factors mentioned above, the radiation shield should be optimized so that sum of these two factors is as minimal as possible, as formulated in (4). These optimizations process is performed by considering the effects of total ionizing dose, secondary particles, and the number of shield layers. The common material in space hardware is aluminum as both a radiation shield and structural enclosure, therefore the designed multi-layered shields are compared with 2 mm thickness of Al.

**Figure 4:**
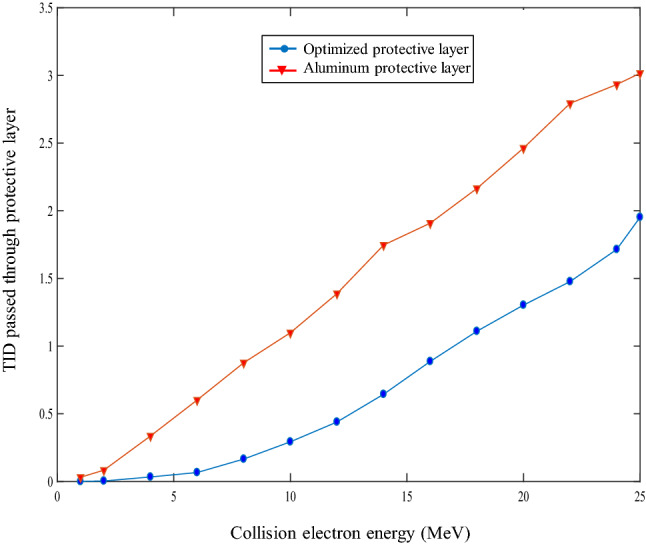
Curve of total ionizing dose passing through optimal shield and a 2 mm aluminum shield in different energies (the blue curve is related to a three-layer shield).

The results of these optimizations can be seen in Table 2 for all three optimized shields. The convergence of the Genetic Algorithm for the three-layer shield is shown in Fig. 4, where combinations of high and low-density materials are achieved in the three cases.

**Table 2:** Results of Genetic Algorithm for three, five, and seven-layer shields.

Shield types	Specifications	Layers						
		1	2	3	4	5	6	7
Three-layers	Material	Gold	Tungsten	Aluminium				
	Thickness (mm)	1.345	0.338	0.300				
Five-layers	Material	Gold	Gold	Tungsten	Titanium	Titanium		
	Thickness (mm)	0.205	0.851	0.729	0.190	0.010		
Seven-layers	Material	Tungsten	Tantalum	Gold	Tantalum	Tungsten	Titanium	Aluminium
	Thickness (mm)	0.350	0.193	0.478	0.474	0.270	0.179	0.029

The properties of these three optimized shields from TID and SP point of view are presented in Table 3. The results of TID and SP are shown in this Table are the normalized output of Tally in MCNPX code and are provided for comparison only. Therefore, the unit is considered as arbitrary (a.u.). According to Table 3, it can be seen that the total ionizing dose is not significantly different in all three states. In the seven-layer model, although the dosage is slightly improved, the fabrication cost is increased compared with the three-layer one. In the construction of multi-layer shields, more layers require more costs and on the other hand, the fabrication would be more complicated technically.

**Table 3:** Specifications of multi-layer radiation shields and aluminum.

Shield types	Total ionizing dose (a.u.)		Secondary particles (a.u.)	Total thickness (mm)
	Value	Percentage		
Three-layer	$0.5130\times {10}^{-6} \pm 0.03\%$	32	$0.6129\times {10}^{-5} \pm 0.02\%$	1.983
Five-layer	$0.5986\times {10}^{-6} \pm 0.05\%$	38	$0.4071\times {10}^{-5} \pm 0.04\%$	1.985
Seven-layer	$0.4524\times {10}^{-6} \pm 0.06\%$	28	$0.4721\times {10}^{-5} \pm 0.03\%$	1.973
Aluminium	$1.55713\times {10}^{-6} \pm 0.03\%$	100	$0.4013\times {10}^{-5} \pm 0.02\%$	2

Now that we have come up with an optimal design, and need to evaluate this optimization from various aspects. The designed shield must be capable of repelling radiation effects across all energy ranges and operating better than an Aluminum shield with the same thickness (2 mm) in all conditions of electron energy.

The total ionizing dose passing through the designed three-layer and the Aluminum is compared and plotted for electron sources with different energies in Fig. 4. It can be seen that the optimized three-layer dose is almost 70% better than the single Aluminum layer, meaning it can meet the satellite requirement and has a significant advantage over all different electron energy ranges. The radiation dose is defined as the energy deposited in the material, the unit of this quantity is the ratio of energy to the mass of material (jerk/g). 1 jerk = 1 GJ (Giga Joule).

### High dose environment

**Figure 5:**
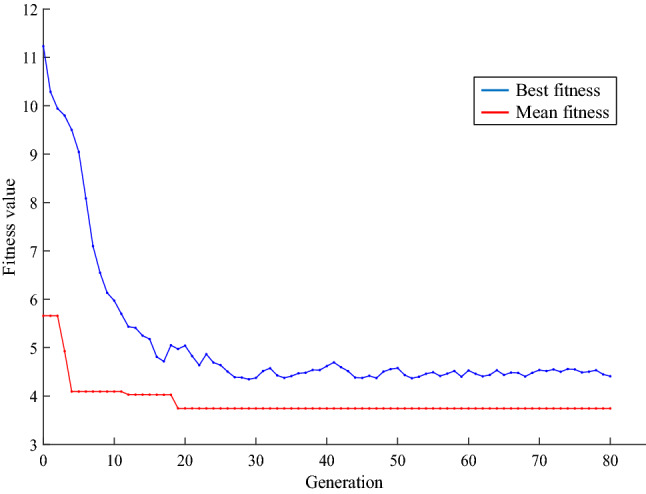
Convergence curve of GA for three-layer radiation shielding applied for proton space environments.

In this section, optimization and analysis of radiation shielding for proton environments are discussed. The designed shields are optimized for the worst space conditions, which can be used in any space environment. For this purpose, the proton energy is considered in the range of 1 to 100 MeV^41^. The optimization process is performed to provide a suitable protection structure for proton environments optimized in terms of ionization dose, secondary particles, and the number of layers. The results of Genetic Algorithm optimization for three, five, and Seven-layer shields are shown in Table 4. The cost function values versus GA iterations for three-layer case are also shown in Fig. 5, which illustrates the algorithm convergence with the three-layer shield.

**Table 4:** The results of the GA for three, five, and seven-layer shields.

Shield types	Specifications	Layers						
		1	2	3	4	5	6	7
Three-layers	Material	Tungsten	Lead	Tantalum				
	Thickness (mm)	0.705	0.589	0.703				
Five-layers	Material	Gold	Tantalum	Gold	Copper	Copper		
	Thickness (mm)	0.478	0.509	0.211	0.302	0.500		
Seven-layers	Material	Tantalum	Tungsten	Tantalum	Tantalum	Tungsten	Tungsten	Lead
	Thickness (mm)	0.400	0.352	0.0910	0.393	0.299	0.350	0.108

Table 5 shows the functional properties of the optimized multi-layer shields and Aluminum shield for comparison. The total ionizing dose at the five and Seven-layer shields are not significantly differ from Three-layer shield, in which only slightly improved at the Seven-layer shield. Since protons generally produce fewer secondary particles, they tend to have a minimum value acceptable for the all three shields. Therefore, a Three-layer shield can be selected as a good enough low-cost shield layer. By comparing the total ionizing dose in the Three-layer shield and the Aluminum one, it can be found out that the Three-layer shielding optimizes the total ionizing dose up to 50%, and also Three-layer shield performs much better than the usual 2 mm Aluminum shield regarding the producing less secondary particles.

**Table 5:** Typical specifications of multi-layer radiation and Aluminum shields.

Shield types	Total ionizing dose (a.u.)		Secondary particles (a.u.)	Total thickness (mm)	Fabrication process cost
	Value	Percentage			
Three-layer	2.6926 × ${10}^{-6}$	50	0.0196 × ${10}^{-7}$	1.997	Low
Five-layer	2.7066 × ${10}^{-6}$	50	0.0356 × ${10}^{-8}$	2	Medium
Seven-layer	2.4867 × ${10}^{-6}$	46.7	0.0116 × ${10}^{-5}$	1.993	High
Aluminium	5.3142 × ${10}^{-6}$	100	0.0458 × ${10}^{-5}$	2	Low

**Figure 6:**
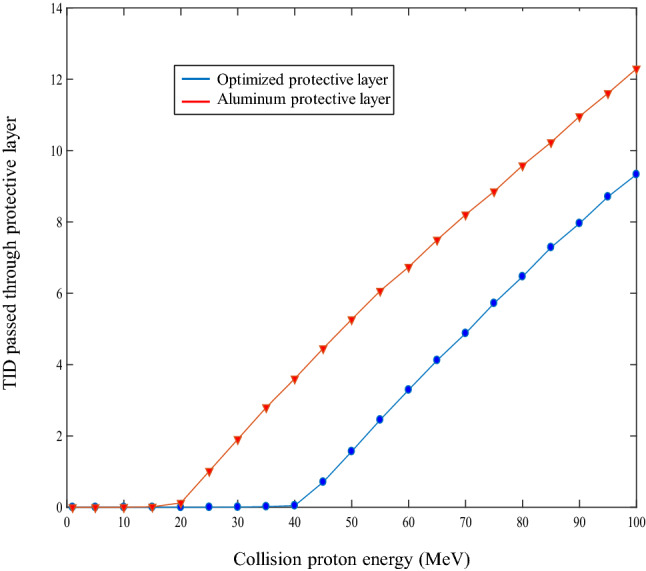
Total ionizing dose versus particle energy for optimized three-layer and Aluminum shield (the blue curve is related to a three-layer shield).

In Fig. 6, the total ionizing dose pass of shielding and Aluminum layers for the proton sources in different energy ranges are compared and drawn, as shown in Fig. 6. The optimized layer is 50% better than the single Aluminum layer over most of the cases.

### Design to build

Due to the lack of access to all materials and economic conditions, a real three-layer shield case is considered by using COTS materials reported in Table 6 for demonstration.

**Table 6:** The COTS materials used in the optimization process of manufactured radiation shields.

Number	Material	Density
1	Copper	8.94
2	Molybdenum	10.28
3	Aluminium	2.7
4	Tin bronze	8.78
5	Bronze aluminium	8.316

**Figure 7:**
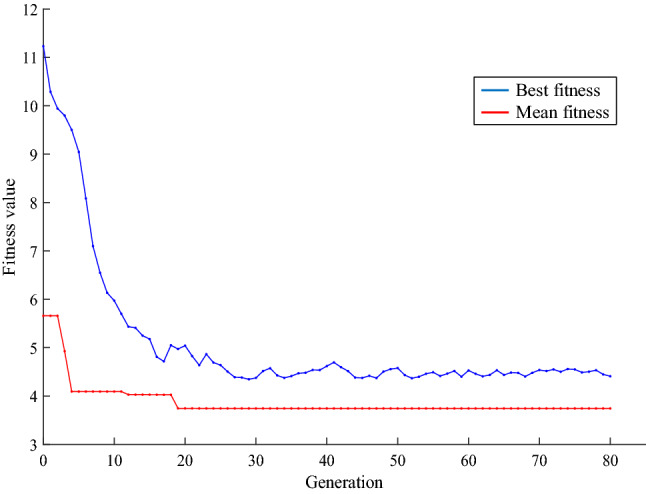
Convergence curve of Genetic Algorithm for constructed shield.

A copper layer with 0.2 mm thickness is considered as the shield carrier for technical implementation reasons. Therefore, the other total layer thickness is considered as 1.8 mm. Due to the significant importance of proton damage in the LEO satellite, the study is focused on using this source in the energy range of 1–100 MeV. The optimization results for different layers indicate that the best case is Three-layer shield to interaction with proton. The results of this optimization are shown in Table 7. The specifications of the first, second, and third layers are presented in this table, respectively. Notice that the zero layer is related to 0.2 mm copper carrier. The convergence process of the Genetic Algorithm is also shown in Fig. 7.

**Table 7:** specifications of optimized three-layer shield.

Layers	Materials	Thickness (mm)	Density (g/cm^3^)
Layer-1	Bronze aluminum	0.795	8.316
Layer-2	Molybdenum	0.629	10.28
Layer-3	Bronze aluminum	0.318	8.316

## Fabrication and test

**Figure 8:**
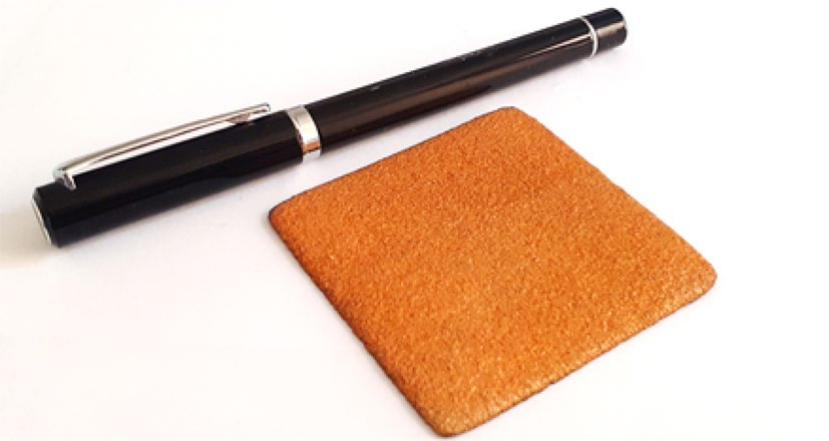
Fabricated three-layer radiation shield sample.

The three-layer shield is implemented by using spotting metal method on 0.2 thickness copper sheet as discussed previously. The constructed shield sample with 5 cm × 5 cm size is depicted in Fig. 8.

**Figure 9:**
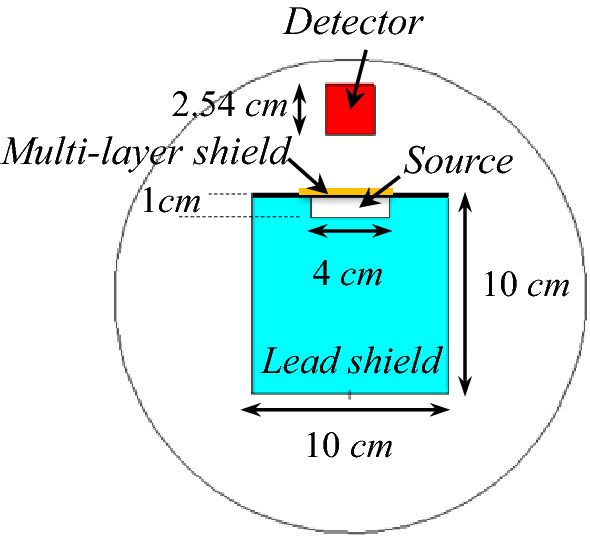
Configuration of the source, detector and shield in the MCNPX code.

**Figure 10:**
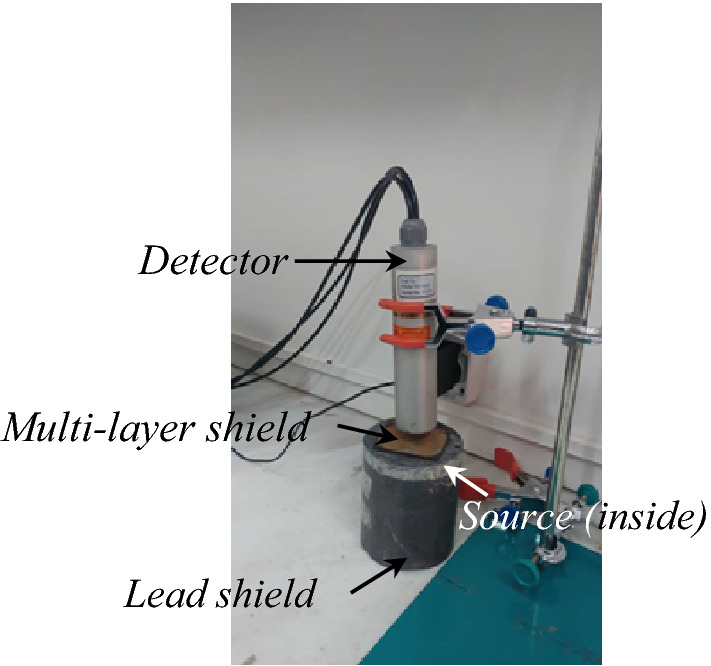
Configuration of irradiation measurement setup using a CsI (Tl) detector (with 1″ × 1″ size of scintillator crystal).

The measurements are performed using the CsI (Tl) scintillator detector model NT-812 as shown in Fig. 10. The MCNPX code is used to determine the attenuation coefficient through the simulation. “By having the flux in the presence and absence of the radiation shield using (1), the radiation attenuation coefficient is obtained. The radiation attenuation coefficient is also calculated by using MCNPX code. In the MCNPX simulation, the arrangement of the detector (Fig. 9), radiation shield and source is exactly same as the experimental procedures arrangement in the laboratory according to Fig. 10. This is done to validate the simulation results in the optimal shield design using the MCNPX code.

In the experimental phase, the CsI (Tl) scintillator detector is applied regarding the higher efficiency for detection the gamma rays in comparison with the similar detectors like NaI (Tl), according to the experimental work, and. F2 Tally in the MCNPX is used to determine the flux. The radiation sources with different gamma energies tabulated in Table 8 are used for irradiation of the proposed shield, also. The configuration of this measurement is shown in Fig. 10 in which multiple shields are placed in front of the source and read by the detector.

**Table 8:** Gamma radioisotope sources for irradiation.

Source	Am-241	Ba-133		Co-57	Na-22		Cs-137	Co-60	
Energy (keV)	59	80	356	122	511	1275	662	1173	1333

**Figure 11:**
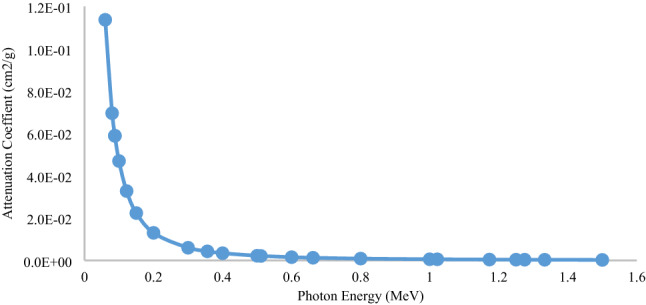
Mass attenuation coefficient of Aluminum Bronze using the XCOM.

The XCOM program is also used to determine the radiation attenuation coefficient of these different layers. These results are taken into account by considering the coherence and non-coherence distribution, photoelectric effects, and pair production. The results of the Aluminum Bronze mass attenuation coefficient can be seen in Fig. 11 as representative. The results for other materials can be extracted in the same way.

### Determination of the attenuation coefficient of multi-layer shield using XCOM

To determine the radiation attenuation coefficient, we assume B = 1, therefore, by using (1), the radiation attenuation coefficient can be calculated by the experimental methods and Monte Carlo simulations. Since multi-layered shield is not defined in XCOM software, so the quantity µx namely the radiation attenuation coefficient multiplied by the thickness is used to compare the results. In this case, this quantity as the same as µx in (3) is obtained by

5 $$\upmu x =\upmu _{1} {\text{x}}_{1} +\upmu _{2} {\text{x}}_{2} +\upmu _{3} {\text{x}}_{3} + ... $$

**Figure 12:**
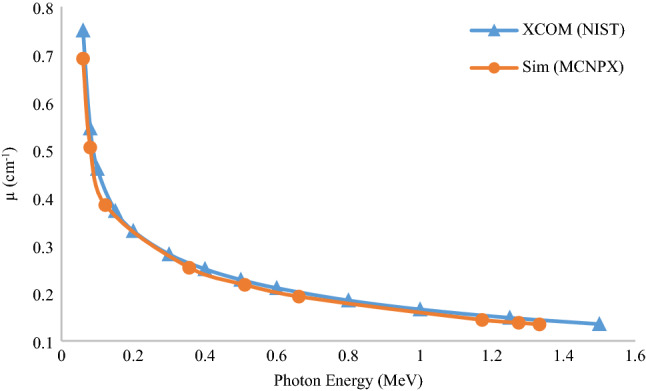
Comparison of attenuation coefficient of Aluminium extracted from XCOM and MCNPX.

For an approximated validation of the data, the radiation attenuation coefficients obtained from the XCOM program and the MCNPX output can be compared. These results are performed for a 2 mm Aluminum shield. The geometric arrangement used for the shield positions, source, and detector are the same as the multi-layered shield arrangement designed to be placed in front of the gamma source, which has also been performed, experimentally. The results are shown in Fig. 12.

Table 9 shows the multiplication of the attenuation coefficient of the constituent materials in thickness or, in other words, μx at different energies. The radiation attenuation coefficient is obtained by using the online program XCOM under NIST.

**Table 9:** Multiplication of radiation attenuation coefficient of multi-layered shield and thickness using XCOM.

Energy (MeV)	Layer -1 BzAl795	Layer-3 BzAl318	Layer-2 Mo	Layer-0 Cu	µx total
0.06	1.135	0.454	2.764	0.357	4.710
0.08	0.543	0.217	1.269	0.171	2.200
0.1	0.360	0.144	0.709	0.082	1.295
0.122	0.239	0.096	0.434	0.069	0.838
0.15	0.166	0.067	0.272	0.040	0.545
0.2	0.112	0.045	0.157	0.028	0.342
0.3	0.077	0.031	0.089	0.020	0.217
0.356	0.069	0.027	0.075	0.023	0.193
0.4	0.064	0.026	0.068	0.017	0.174
0.5	0.056	0.022	0.057	0.015	0.151
0.511	0.056	0.022	0.056	0.019	0.153
0.6	0.051	0.020	0.051	0.014	0.136
0.662	0.048	0.019	0.048	0.016	0.132
0.8	0.044	0.018	0.043	0.012	0.116
1	0.039	0.016	0.038	0.011	0.103
1.022	0.039	0.015	0.037	0.010	0.102
1.173	0.036	0.014	0.035	0.012	0.097
1.25	0.035	0.014	0.033	0.009	0.092
1.275	0.035	0.014	0.033	0.012	0.093
1.333	0.034	0.014	0.032	0.011	0.091
1.5	0.032	0.013	0.030	0.009	0.084

**Figure 13:**
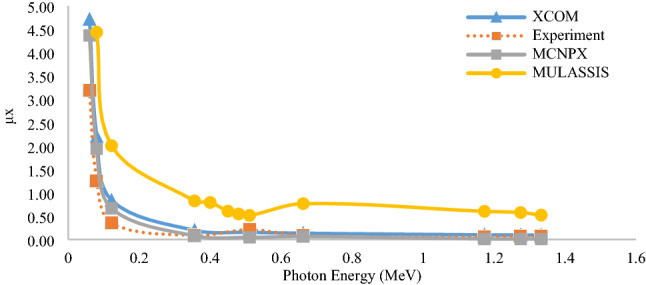
Comparison of radiation attenuation coefficient of multi-layer shield obtained from experimental, analytical and simulation results.

The results of obtaining the attenuation coefficient of a multi-layer shield are depicted in Fig. 13 via various approaches, including experimental, analytical, and simulation methods. As shown, the output results related to the XCOM program and the MCNPX code in the energy range of 0.06–1.5 MeV exhibited a good agreement. Therefore, the results of this comparison are performed for a multi-layer radiation shield. The radiation attenuation coefficient obtained from the experimental data, XCOM analysis method, Monte Carlo MCNPX code, and MULASSIS tool are shown in Fig. 13 for gamma rays.

As shown in Fig. 13, the output of the experimental results, XCOM program, and the MCNPX are closer to each other, and the values obtained from the MULASSIS tool differ from the obtained values. The significant difference in the results of this tool with the other methods is related to the very low accuracy of particle transport. This code is used as a module in SPENVIS web-based software and is limited in the number of transport particles. The method of transportation in this code is Monte Carlo and random, so if the number of transport particles is reduced, the statistical error is increased; thus, this is one of the critical reasons for the significant difference in results with the other methods. Another reason for the discrepancy could be the difference in cross-sections used in the libraries. In simulation and statistical methods, the sources are considered single energy, while there are no single energy sources in practice, and the source has a spectrum of energy. Also, the existence of unknown dispersions from the laboratory environment, the detection accuracy, and the presence of many impurities in the multi-layer shield are the main reasons for the difference in computational data with the experimental results. The difference in MCNPX code results and XCOM program can be due to the differences in cross-section and differences in the geometric arrangement, source, protection, and detector. In general, differences can be due to the presence of different layers in the design and construction of the shield. The presence of these layers complicates analysis and simulation, and analysis is not as easy as using single-layer shields. Due to the high consistency of the experimental and simulation results using the MCNPX code, this trend can also be generalized to similar cases.

Since the design and construction of multi-layer shield is based on the simulation of this code, and according to the results of the nuclear engineering designs that have been done with this code, it can be deduced that the simulation of the built-in shield is suitable for using local shield and safety of electronic components of satellites.

## Conclusions

In this paper, various types of multi-layer shields in space conditions were designed, optimized, and analyzed for electron and proton environments. This is done for various suitable metals to be used as a local shield for the safety of electronic components, using the MCNPX Monte Carlo method and the Genetic Algorithm. These designed shields were discussed at different energies in all space conditions and compared with 2 mm thickness of Aluminum shields, which revealed the complete superiority of designed shields optimized for electrons up to 70% improve the total ionizing dose, and the shield designed for the proton also improved the total ionizing dose up to 50%. All designed shields exhibited the potential to reduce the effects of secondary radiations. Optimizations were made for three, five, and seven-layer shields. Moreover, the Three-layer shield showed advantages over traditional shields such as Aluminum due to the diversity and lower number of layers, and lower construction cost. By imposing restrictions on the construction of the shield, the ultimate optimal case is a combination of three layers of Bronze- Aluminum and Molybdenum. To validate the simulation results, it is necessary to protect satellite tools against the proton shield with a maximum energy of 100 MeV. In this work, due to the limitations of using proton sources, gamma radioisotope sources with an energy range of 60–1333 keV were used for irradiation. Then, the validation of experimental results and calculations was carried out using the MCNPX code. The results indicate a good correlation between the experimental data, simulation, and analytical calculations using the MCNPX code and the XCOM program. Therefore, this subject can be generalized to the other similar cases. Since the design and construction of a multi-layer radiation shield is based on the simulation of MCNPX code, and according to the results of the nuclear engineering designs have been carried out using this code, it can be concluded that the simulation of the built-in shield is suitable for using a local shield and safety of electronic components of the satellites.
